# Amantadine delayed release/extended release capsules significantly reduce OFF time in Parkinson’s disease

**DOI:** 10.1038/s41531-022-00291-1

**Published:** 2022-03-18

**Authors:** Robert A. Hauser, Judy Lytle, Andrea E. Formella, Caroline M. Tanner

**Affiliations:** 1 University of South Florida, Tampa, FL USA; 2 Adamas Pharmaceuticals, Inc, Emeryville, CA USA; 3 UC San Francisco, San Francisco, CA USA

**Keywords:** Parkinson's disease, Parkinson's disease

## Abstract

Maintaining consistent levodopa benefits while simultaneously controlling dyskinesia can be difficult. Recently, an amantadine delayed release/extended release (DR/ER) formulation (Gocovri^®^) indicated for dyskinesia received additional FDA approval as an adjunct to levodopa for the treatment of OFF episodes. We evaluated OFF time reductions with amantadine-DR/ER in a pooled analysis of two phase III amantadine-DR/ER trials (NCT02136914, NCT02274766) followed by a 2-year open-label extension trial (NCT02202551). OFF outcomes were analyzed for the mITT population, as well as stratified by baseline OFF time of ≥2.5 h/day or <2.5 h/day. At Week 12, mean placebo-subtracted treatment difference in OFF time was −1.00 [−1.57, −0.44] h in the mITT population (*n* = 196), −1.2 [−2.08, −0.32] h in the ≥2.5 h subgroup (*n* = 102) and −0.77 [−1.49, −0.06] in the <2.5 h subgroup (*n* = 94). Amantadine-DR/ER-treated participants showed reduced MDS-UPDRS Part IV motor fluctuation subscores by week 2 that were maintained below baseline to Week 100.

## Introduction

Levodopa-induced motor complications (e.g. OFF episodes and dyskinesia) remain a common barrier to the effective management of Parkinson’s disease (PD). In patients with motor complications, maintaining consistent therapeutic efficacy through the day while simultaneously trying to control dyskinesia can be difficult with dopaminergic therapy. Despite advances in our understanding of the pathophysiology and risk factors for their development^1–3^, over 50% of people using levodopa experience OFF episodes, dyskinesia or both within 5 years, and up to 100% after 10 years following PD diagnosis^4,5^.

Of all the medications available to manage PD, only amantadine has established efficacy against levodopa-induced dyskinesia^6,7^. Early studies with immediate-release amantadine were plagued by inconsistent results, and while higher doses were associated with greater efficacy, they were also associated with worse tolerability. To address these issues, a delayed release/extended release (DR/ER) capsule formulation of amantadine (ADS-5102; Gocovri^®^ [amantadine] ER capsules, Adamas Pharmaceuticals, Emeryville, CA) was specifically developed as a once-daily, bedtime-administered formulation, containing coated pellets that provide a reliable delivery profile, with an initial delay in exposure, followed by sustained amantadine delivery over the dosing interval. In contrast to other immediate release (IR) and ER formulations, amantadine-DR/ER, has consistently shown sustained and clinically meaningful efficacy against dyskinesia across well-controlled clinical trials, resulting in the product receiving FDA approval for treatment of levodopa-related dyskinesia in Parkinson’s disease in 2017^8–10^. Even though these trials recruited patients with dyskinesia (i.e. there was no minimum requirement for OFF time), significant improvement in OFF time was also observed, with an overall placebo-adjusted reduction of 1 h, representing an ~36% reduction in OFF time from baseline^8–11^. These findings led to the FDA recently granting amantadine-DR/ER an additional indication as adjunctive treatment to levodopa for the management OFF episodes^12^.

To further explore the effects of amantadine-DR/ER to reduce OFF time, we evaluated OFF time measures in the pooled pivotal trials as well as in the 2-year open-label extension trial. Treatment trials for OFF episodes as a primary outcome typically enroll patients with 2 to 3 h of OFF time/day. Therefore, in addition to the overall study cohort, we analyzed participants stratified by baseline OFF time of ≥ or < 2.5 h per day (the median for our sample) to specifically analyze the subgroup of participants who would meet eligibility criteria for an interventional trial aimed at reducing OFF time, as well as the subgroup with less OFF time at baseline.

## Results

### Participant disposition and baseline characteristics

**Fig. 1:**
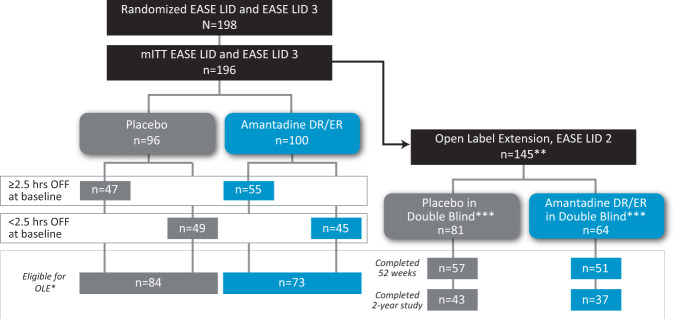
Patient disposition. *Completed double-blind trial or were active in EASE LID which was stopped early by the sponsor to accelerate data submission to the US Food and Drug Administration^9^. All randomized patients had the opportunity to complete their week 12 trial visit (time of primary efficacy assessment). **An additional 78 patients (not included in the analyses) were enrolled, *n* = 17 who participated in a phase II dose-finding trial and later enrolled in EASE LID 2 after a gap in amantadine-DR/ER therapy, and *n* = 61 who had DBS and did not participate in double-blind trials^41^. *** Seven participants (*n* = 3 who received placebo and *n* = 4 who received amantadine-DR/ER in double-blind pivotal trials experienced a gap in treatment before entering the open-label trial. For purposes of these analyses we have included them with their original double-blind treatment groups.

Of the 198 randomized participants across both double-blind trials^9,10^, 196 were included in the mITT population and their OFF time was analyzed. Of these, 102 participants had ≥2.5 h of OFF time at baseline and 94 participants had <2.5 h of OFF time at baseline, 23 of whom had 0 h OFF (Fig. 1). Overall, 145 participants included in the mITT population of the pooled pivotal trial dataset transitioned to the extension trial and were included in the open-label analyses. Of these, 80 completed the open-label extension trial.

Participant characteristics are summarized in Table 1. Overall, 173 of 196 (88%) participants reported experiencing OFF time on their home diaries at baseline. Mean daily OFF time was 2.8 h in the mITT population, 4.4 h in the ≥2.5 h subgroup and 1.0 h in the <2.5 h subgroup (including the 23 participants with 0 h of OFF time at baseline).

**Table 1:** Baseline characteristics.

Variable	mITT (*N* = 196)	Baseline OFF ≥ 2.5 h (*n* = 102)	Baseline OFF < 2.5 h (*n* = 94)
Age (years), mean ± SD	64.7 ± 9.2	62.9 ± 9.1	66.7 ± 8.8
Sex, *n* (%) male	109 (55.6)	54 (52.9)	55 (58.5)
Race, *n* (%)			
White	185 (94.4)	96 (94.1)	89 (94.7)
Other	11 (5.6)	6 (5.9)	5 (5.3)
Duration of levodopa treatment (years), mean ± SD	7.7 ± 4.0	8.0 ± 4.0	7.3 ± 3.9
Duration of dyskinesia (years), mean ± SD	3.8 ± 2.8	4.1 ± 3.1	3.4 ± 2.6
Levodopa dose (mg), mean ± SD	782 ± 491	776 ± 476	789 ± 508
LEDD (mg), mean ± SD	1024 ± 541	1026 ± 514	1021 ± 573
Concomitant PD medications, n (%)			
Any Dopamine agonist/MAOB/COMT inhibitor^1^	142 (72.4)	75 (73.5)	67 (71.3)
Dopamine agonist	106 (54.1)	60 (58.8)	46 (48.9)
MAOB-inhibitor	86 (43.9)	39 (38.2)	47 (50.0)
COMT inhibitor*	74 (37.8)	36 (35.3)	38 (40.4)
Anticholinergic	7 (3.6)	3 (2.9)	4 (4.3)
OFF time (h)			
Mean ± SD	2.8 ± 2.1	4.4 ± 1.6	1.0 ± 0.8
Median [range]	2.5 [0–9.5]	4.0 [2.5–9.5]	1.0 [0–2.3]
ON time without troublesome dyskinesia (h); mean ± SD	8.4 ± 3.1	7.3 ± 2.7	9.5 ± 3.1
ON time with troublesome dyskinesia (h); mean ± SD	4.9 ± 2.6	4.6 ± 2.2	5.2 ± 2.9
MDS-UPDRS			
Total score (Parts 1-3)	50.5 ± 18.0	51.5 ± 19.1	49.4 ± 16.7
MDS-UPDRS Part 1 score	11.4 ± 5.4	11.5 ± 5.1	11.4 ± 5.7
MDS-UPDRS Part 2 score	15.2 ± 6.2	15.8 ± 6.3	14.6 ± 6.2
MDS-UPDRS Part 3 score	23.8 ± 12.2	24.2 ± 14.1	23.4 ± 9.9
MDS-UPDRS Part 4 score	11.1 ± 2.7	11.7 ± 2.2	10.6 ± 3.1
MDS-UPDRS Part 4 OFF items (4.3 to 4.6)	6.1 ± 2.5	6.8 ± 1.9	5.4 ± 2.9
MDS-UPDRS Part 4 Dyskinesia items (4.1 + 4.2)	5.0 ± 1.1	4.9 ± 1.0	5.2 ± 1.1
UDysRS Total score	40.1 ± 12.1	41.2 ± 11.8	39.0 ± 12.4

^1^Including COMT inhibitor combinations e.g., levodopa, carbidopa, entacapone combination (Stalevo).

*LEDD* levodopa equivalent daily dose, *MAOB* monoamine oxidase B, *COMT* Catechol-O-Methyl-Transferase, *MDS-UPDRS* Movement Disorder Society - Unified Parkinson’s Disease Rating Scale; The MDS-UPDRS has four parts: Part I (non-motor experiences of daily living), Part II (motor experiences of daily living), Part III (motor examination) and Part IV (motor complications). *UDysRS* Unified Dyskinesia Rating Scale.

### Efficacy in pooled double-blind trials

**Fig. 2:**
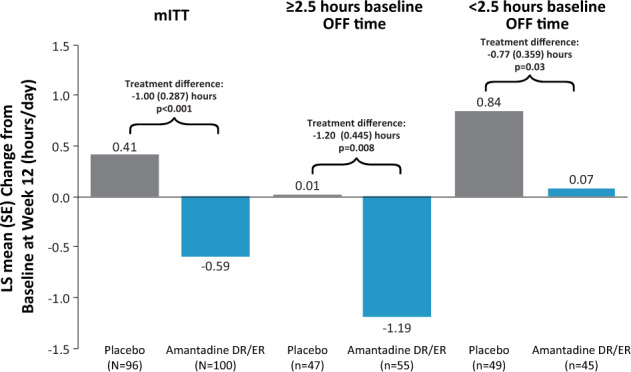
LS Mean (SE) Change from Baseline to Week 12 in OFF time. Data are for the mITT population and stratified by baseline OFF time (≥2.5 h and < 2.5 h subgroups). *P*-values are based on comparisons between amantadine DR/ER versus placebo from an MMRM model with change from baseline as the dependent variable and baseline values as a covariate. The model includes categorical effects for treatment group, study, and visit (weeks 2, 8, and 12), and the interaction between treatment group and visit.

As shown in Fig. 2, 12 weeks’ treatment with amantadine-DR/ER significantly reduced OFF time relative to placebo in all treatment groups (Table 2). In the ≥2.5 h subgroup, participants treated with amantadine DR/ER experienced a mean [95% CI] placebo-subtracted reduction in OFF time from baseline to Week 12 of 1.2 [−2.1, −0.3] h (*p* = 0.008). In the <2.5 h subgroup, participants treated with amantadine DR/ER experienced a placebo-subtracted reduction in OFF time from baseline to Week 12 of 0.8 [−1.5, −0.1] h vs. placebo (*p* = 0.03).

**Table 2:** Changes from baseline in time spent in OFF, ON without troublesome dyskinesia, and ON with troublesome dyskinesia diary states, mITT population and by baseline OFF time (MMRM analysis).

Variable	mITT (*n* = 196)^8^			Baseline OFF ≥ 2.5 h (*N* = 102)			Baseline OFF < 2.5 h (*N* = 94)		
	Placebo (N = 96)	Amantadine-DR/ER (*N* = 100)	LS Mean [95% CI] Treatment difference	Placebo (N = 47)	Amantadine-DR/ER (*N* = 55)	LS mean [95%CI] Treatment difference	Placebo (N = 49)	Amantadine-DR/ER (*N* = 45)	LS Mean [95% CI] Treatment difference
Change from baseline in OFF time (h); LS mean ± SE									
Week 2	−0.2 ± 0.2	−0.5 ± 0.2	−0.3 [−0.8, 0.1]	−0.6 ± 0.3	−1.1 ± 0.3	−0.5 [−1.2, 0.2]	0.2 ± 0.2	0.1 ± 0.2	−0.1 [−0.6, 0.4]
Week 8	0.2 ± 0.2	−0.5 ± 0.2	−0.7 [−1.2, −0.2]	−0.2 ± 0.3	−1.0 ± 0.3	−0.8 [−1.7, 0.0]	0.6 ± 0.2	0.1 ± 0.2	−0.5 [−1.1, 0.1]
Week 12	0.4 ± 0.2	−0.6 ± 0.2	−1.0 [−1.6, −0.4]	0.0 ± 0.3	−1.2 ± 0.3	−1.2 [−2.1, −0.3]	0.8 ± 0.2	0.1 ± 0.3	−0.8 [−1.5, −0.1]
Change from baseline ON time without troublesome dyskinesia (h); LS mean ± SE									
Week 2	1.5 ± 0.3	3.8 ± 0.3	2.3 [1.5, 3.1]	1.5 ± 0.5	4.1 ± 0.4	2.6 [1.4, 3.8]	1.3 ± 0.4	3.4 ± 0.4	2.2 [1.0, 3.3]
Week 8	1.9 ± 0.4	4.1 ± 0.4	2.2 [1.2, 3.2]	1.3 ± 0.5	4.4 ± 0.5	3.1 [1.7, 4.5]	2.1 ± 0.5	3.6 ± 0.5	1.5 [0.2, 2.9]
Week 12	1.4 ± 0.3	3.8 ± 0.4	2.4 [1.5, 3.4]	0.7 ± 0.5	4.1 ± 0.5	3.4 [2.2, 4.7]	1.8 ± 0.5	3.4 ± 0.5	1.6 [0.2, 2.9]
Change from baseline ON time with dyskinesia (h); LS mean ± SE									
Week 2	−1.2 ± 0.4	−3.7 ± 0.4	−2.6 [−3.6 −1.6]	−0.9 ± 0.5	−3.1 ± 0.5	−2.2 [−3.6, −0.8]	−1.4 ± 0.5	−4.4 ± 0.5	−3.0 [−4.5, −1.5]
Week 8	−2.1 ± 0.4	−4.0 ± 0.4	−1.9 [−3.0, −0.7]	−1.2 ± 0.6	−3.4 ± 0.5	−2.1 [−3.6, −0.7]	−3.0 ± 0.6	−4.7 ± 0.7	−1.7 [−3.5, 0.0]
Week 12	−2.2 ± 0.4	−4.1 ± 0.4	−1.9 [−3.1, −0.7]	−1.5 ± 0.6	−3.6 ± 0.5	−2.1 [−3.6, −0.6]	−2.9 ± 0.6	−4.7 ± 0.7	−1.8 [−3.6, 0.1]
Change from baseline ON time with troublesome dyskinesia (h); LS mean ± SE									
Week 2	−1.4 ± 0.3	−3.3 ± 0.3	−1.8 [−2.5, −1.1]	−1.1 ± 0.4	−3.0 ± 0.3	−1.9 [−2.9, −1.0]	−1.7 ± 0.4	−3.4 ± 0.4	−1.7 [−2.7, −0.7]
Week 8	−2.1 ± 0.3	−3.6 ± 0.3	−1.5 [−2.2, −0.7]	−1.5 ± 0.4	−3.3 ± 0.3	−1.8 [−2.7, −0.8]	−2.5 ± 0.4	−3.8 ± 0.4	−1.2 [−2.4 −0.1]
Week 12	−1.9 ± 0.3	−3.3 ± 0.3	−1.5 [−2.3, −0.7]	−0.9 ± 0.4	−2.9 ± 0.4	−2.0 [−3.1, −1.0]	−2.6 ± 0.4	−3.6 ± 0.4	−1.0 [−2.2 0.2]

Treatment difference and confidence intervals are based on MMRM model with change from baseline as the dependent variable and baseline as a covariate. The model includes categorical effects for treatment group, study, and visit (weeks 2, 8, and 12), and the interaction between treatment group and visit.

For the ≥2.5 h subgroup, whereas treatment with placebo was associated with an initial LS mean decrease in OFF time (−0.6 h) at week 2 that reverted to baseline over subsequent visits, participants treated with amantadine-DR/ER showed an LS mean reduction in OFF time >1 h versus baseline by Week 2, that was maintained at subsequent visits. In the <2.5 h subgroup, whereas participants treated with placebo showed a progressive increase in OFF time (from 1.0 h at baseline to 1.8 h at Week 12), there was no increase in mean OFF time for participants treated with amantadine-DR/ER (1.0 h at baseline and Week 12). Of the 11 amantadine-DR/ER-treated participants who recorded zero hours of OFF time at baseline, 7 maintained zero hours OFF time at week 12. Conversely, 8 of the 12 placebo-treated participants who recorded no OFF time at baseline had reported OFF time at Week 12 or study drop-out (4 maintained zero hours OFF time at week 12). A responder analysis demonstrated greater response rates for participants receiving amantadine DR/ER versus placebo. For example, of participants reporting OFF time at baseline, 54% taking amantadine DR/ER experienced at least a 25% OFF time reduction vs. 37% for placebo, and 18% vs. 11% reported a 100% reduction (Supplemental Table e1).

Reductions in OFF time were accompanied by significant increases in ON time without troublesome dyskinesia versus placebo (Table 2). At Week 12, the placebo-subtracted treatment differences were 2.4 h in the mITT population (*p* < 0.001)^8^, 3.4 h in the ≥2.5 h subgroup (*p* < 0.001) and 1.6 h in the <2.5 h subgroup (*p* = 0.02). Reductions in OFF time and troublesome dyskinesia were not correlated for amantadine-DR/ER-treated participants; a weak negative correlation (decreases in OFF time with increases in troublesome dyskinesia) was seen for placebo-treated participants (Supplemental Fig. e1).

Consistent with results previously reported for the mITT population pooled analysis^8^, participants in the ≥2.5 h subgroup showed significant improvements in CGI-C (Table 3) and mean [95% CI] Movement Disorder Society–Unified Parkinson’s Disease Rating Scale (MDS-UPDRS)^13^ Part II scores (−3.4 [−5.1, −1.6] vs. placebo). The CGI-C was significantly improved in the <2.5 h subgroup but the MDS-UPDRS Part II was not, and neither subgroup showed significant overall change in MDS-UPDRS Parts I or III. Item scores for MDS-UPDRS Parts I and II have previously been reported for the mITT population^14^ and are reported for the ≥2.5 h subgroup in Supplemental Fig. e2. In the ≥2.5 h subgroup, daytime sleepiness, depression and apathy (MDS-UPDRS Part I) and tremor, speech, eating tasks, getting out of bed/deep chair, freezing, and turning in bed (MDS-UPDRS Part II) showed a significant positive treatment effect, and hallucinations (MDS-UPDRS Part I) showed a negative treatment effect (better with placebo).

**Table 3:** LS Mean (SE) changes from baseline in MDS-UPDRS Part IV subscores, item scores and CGI-C, mITT population and by baseline OFF time (MMRM).

	mITT (*n* = 196)			Baseline OFF ≥ 2.5 h (*N* = 102)			Baseline OFF < 2.5 h (*N* = 94)		
	Placebo (*N* = 96)	Amantadine-DR/ER (*N* = 100)	LS Mean [95% CI] Treatment difference	Placebo (*N* = 47)	Amantadine-DR/ER (*N* = 55)	LS Mean [95% CI] Treatment difference	Placebo (*N* = 49)	Amantadine-DR/ER (*N* = 45)	LS Mean [95% CI] Treatment difference
Clinician’s Global Impression of Change (CGI-C) in Overall PD Symptoms at Week 12, *n* (%)									
Marked Improvement	2 (2.1)	28 (28.0)	–	2 (4.3)	17 (30.9)	–	0	11 (24.4)	–
Moderate Improvement	13 (13.5)	29 (29.0)	–	3 (6.4)	14 (25.5)	–	10 (20.4)	15 (33.3)	–
Minimal Improvement	21 (21.9)	19 (19.0)	–	11 (23.4)	10 (18.2)	–	10 (20.4)	9 (20.0)	–
No Change	40 (41.7)	17 (17.0)	–	20 (42.6)	10 (18.2)	–	20 (40.8)	7 (15.6)	–
Minimal Worsening	17 (17.7)	4 (4.0)	–	9 (19.1)	2 (3.6)	–	8 (16.3)	2 (4.4)	–
Moderate Worsening	3 (3.1)	2 (2.0)	–	2 (4.3)	2 (3.6)	–	1 (2.0)	0	–
*P*-value	*p* < 0.0001			*p* < 0.0001			*p* < 0.0001		
MDS-UPDRS Part IV subscores and item scores, change from baseline at Week 12; LS mean ± SE									
Part IV Total Score	−2.1 ± 0.3	−4.4 ± 0.3	− 2.3 [−3.2, −1.5]	−1.5 ± 0.4	−4.2 ± 0.4	−2.7 [−3.8, −1.6]	−2.6 ± 0.4	−4.7 ± 0.5	−2.1 [−3.4, −0.9]
Dyskinesia items (4.1 + 4.2)	−1.3 ± 0.2	−2.6 ± 0.2	−1.2 [−1.7, −0.7]	−1.1 ± 0.2	−2.3 ± 0.2	−1.2 [−1.9, −0.6]	−1.6 ± 0.3	−2.9 ± 0.3	−1.3 [−2.0, −0.5]
Motor fluctuations items (4.3 + 4.4 + 4.5 + 4.6)	−0.7 ± 0.2	−1.8 ± 0.2	−1.1 [−1.7, −0.4]	−0.3 ± 0.3	−1.8 ± 0.3	−1.5 [−2.2, −0.8]	−1.0 ± 0.4	−1.8 ± 0.4	−0.8 [−1.9, 0.3]
Item 4.1 (time spent with dyskinesia)	−0.6 ± 0.1	−1.0 ± 0.1	−0.5 [−0.8, −0.2]	−0.4 ± 0.1	−0.8 ± 0.1	−0.4 [−0.8, −0.1]	−0.7 ± 0.2	−1.3 ± 0.2	−0.6 [−1.0, −0.1]
Item 4.2 (functional impact of dyskinesia)	−0.8 ± 0.1	−1.5 ± 0.1	−0.8 [−1.0, −0.5]	−0.7 ± 0.2	−1.5 ± 0.1	−0.8 [−1.2, −0.4]	−0.9 ± 0.1	−1.6 ± 0.2	−0.7 [−1.2, −0.3]
Item 4.3 (time spent in the OFF state)	0.0 ± 0.1	−0.3 ± 0.1	−0.3 [−0.5, −0.2]	0.1 ± 0.1	−0.3 ± 0.1	−0.4 [−0.6, −0.2]	0.0 ± 0.1	−0.3 ± 0.1	−0.3 [−0.5, −0.1]
Item 4.4 (functional impact of fluctuations)	−0.4 ± 0.1	−0.8 ± 0.1	−0.4 [−0.7, −0.1]	−0.3 ± 0.2	−0.9 ± 0.2	−0.6 [−1.0, −0.2]	−0.6 ± 0.2	−0.8 ± 0.2	−0.3 [−0.8, 0.2]
Item 4.5 (complexity of motor fluctuations)	−0.2 ± 0.1	−0.3 ± 0.1	−0.1 [−0.3, 0.2]	−0.1 ± 0.1	−0.2 ± 0.1	−0.1 [−0.4, 0.2]	−0.3 ± 0.2	−0.3 ± 0.2	−0.0 [−0.5, 0.4]
Item 4.6 (painful OFF-state dystonia)	−0.1 ± 0.1	−0.4 ± 0.1	−0.3 [−0.6, 0.0]	−0.1 ± 0.2	−0.5 ± 0.1	−0.4 [−0.8, −0.0]	−0.2 ± 0.1	−0.5 ± 0.2	−0.3 [−0.7, 0.1]

*p*-values for CGI-C were calculated using the Cochran–Mantel–Haenszel mean score test using equally spaced scores. Treatment differences for MDS-UPDRS Part IV items are from the MMRM model with change from baseline as the dependent variable and baseline as a covariate, with categorical effects for treatment group, study, visit, and the interaction between treatment group and visit. mITT population.

### Motor complications (MDS-UPDRS Part IV) through the double-blind and open label trials

Consistent with diary data, treatment with amantadine-DR/ER was associated with significant improvements versus placebo in motor complications as assessed by MDS-UPDRS Part IV. Changes in Part IV items and subscores from baseline to Week 12 of the pooled double-blind studies are shown in Table 3. For MDS-UPDRS Part IV, treatment differences (amantadine-DR/ER – placebo) were statistically significant at all double-blind visits for all analyzed sets (mITT population, ≥2.5 h subgroup, and <2.5 h subgroup). When items related to OFF motor fluctuations (sum of 4.3–4.6) were evaluated separately, treatment differences remained significant for the mITT population at all visits, and at Weeks 2 and 12 (endpoint) for the ≥2.5 h subgroup and at Weeks 2 and 8 for the <2.5 h subgroup (Supplemental Table e2). All four motor fluctuation items contributed to the improvements seen with amantadine-DR/ER, with the largest effect versus placebo observed in item 4.4 which assesses the functional impact of motor fluctuations.

**Fig. 3:**
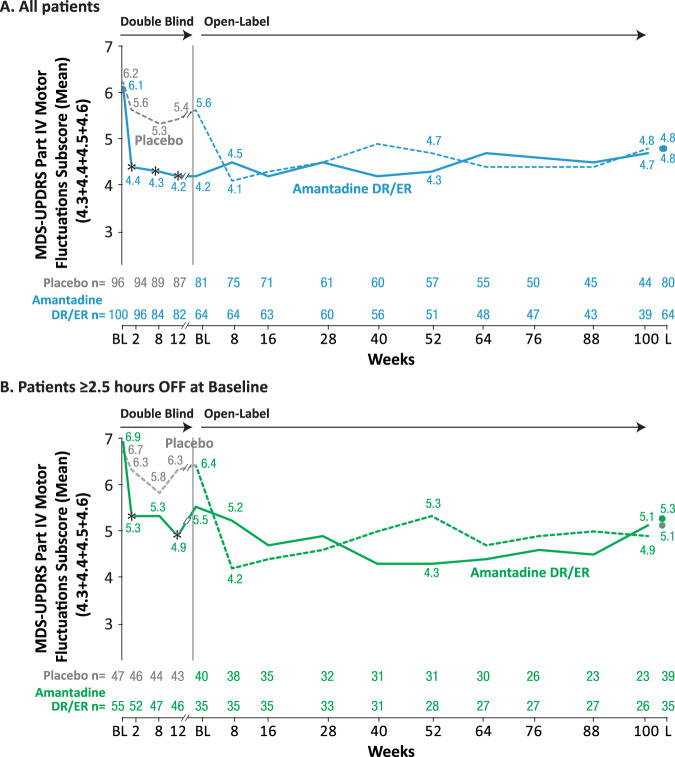
MDS UPDRS Part 4 Motor Fluctuations Subscores through the double-blind and open-label trials for (a) mITT population and (b) ≥ 2.5 h subgroup. Motor Fluctuations Subscore = MDS-UPDRS items 4.3 (time spent in OFF state) +4.4 (functional impact of fluctuations) + 4.5 (complexity of motor fluctuations) +4.6 (OFF state dystonia). For double-blind weeks 2, 8, and 12, *P*-values are based on the comparison between amantadine DR/ER vs. placebo from the MMRM model (*n* = 196 for mITT and *n* = 102 for ≥ 2.5 h subgroup) with change from baseline as the dependent variable and baseline as a covariate, with categorical effects for treatment group, study, and visit (Weeks 2, 8, and 12), and the interaction between treatment group and visit. L = Last on study visit.

During open-label treatment, MDS-UPDRS Part IV scores, including motor fluctuation and dyskinesia subscores, were maintained below baseline levels, at all visits throughout open-label treatment to Week 100, for the mITT, ≥2.5 h and <2.5 h subgroups (Fig. 3). Participants initially treated with placebo showed similar reductions in disability caused by motor fluctuations when they were switched to active treatment with amantadine-DR/ER at the start of the open-label trial, and again, this benefit was maintained out to Week 100.

For the ≥2.5 h subgroup, by open-label Week 100, participants treated with amantadine-DR/ER in double-blind had a mean reduction in motor fluctuation scores of −1.3 points and participants initially treated with placebo and switched to amantadine-DR/ER had a mean reduction of −1.8 points compared to double-blind baseline. (Supplemental Table e3). After 1 and 2 years of treatment, mean MDS-UPDRS Part IV OFF motor fluctuation subscores remained below double-blind baseline regardless of whether patient’s levodopa doses were higher, the same, or lower than at the time of the double-blind baseline.

## Discussion

Based on the consistently statistically significant and clinically meaningful OFF time reductions demonstrated in phase III trials, the FDA has now approved amantadine-DR/ER for adjunctive use with levodopa/carbidopa in persons with Parkinson’s disease experiencing OFF episodes^15^. These reductions in OFF time are in addition to the significant and clinically meaningful reductions in dyskinesia seen in these trials^9,10^, making amantadine-DR/ER the only medication to receive FDA-approval for treating both types of motor complications^12^.

Our analyses confirm and extend these findings, showing that for those patients with significant motor fluctuations at baseline, the magnitude of OFF time reduction is clinically relevant, and at least as large as the reduction associated with other adjunct therapies aimed at managing motor fluctuations. Improvements in diary-recorded OFF time were accompanied by reductions in MDS-UPDRS Part IV motor fluctuation items that were sustained throughout 100 weeks of open-label amantadine-DR/ER treatment, suggesting the benefit in treating OFF episodes is persistent, a finding that was also shown for improvement in dyskinesia items. Importantly, the effect on OFF motor fluctuations in the long-term trial did not appear to result simply from levodopa dose adjustment. Mean MDS-UPDRS OFF items were reduced, even in patient groups taking the same or lower levodopa doses compared to baseline, and were below double-blind baseline at both last-on study visit (which included patients who dropped out) as well as week 100 trial completion.

One of our principal goals was to explore OFF time reductions in a population of patients who were enrolled in a dyskinesia trial, but who would also meet typical entry criteria for trials aimed at reducing motor fluctuations. In patients with ≥2.5 h OFF time at baseline, we observed a significant reduction of 1.2 h OFF time versus placebo, which is greater than that reported in meta-analyses of the COMT inhibitors opicapone (mean reduction of −58.1 minutes OFF time versus placebo)^16^, entacapone (mean reduction of 0.6 h or ∼36 min versus placebo)^17^ or istradefylline (mean reduction of −0.45 h or ∼27 min versus placebo)^18^, drugs that were specifically developed for patients experiencing motor fluctuations. A 1.2 h reduction in OFF time is also considered clinically relevant, as it is above the minimal clinically important difference (MCID) of 1.0 h reported for patients treated with IR pramipexole^19^ and rasagiline^20^. In addition to reduced OFF time, patients in the ≥2.5 h subgroup showed a significant increase in ON time without troublesome dyskinesias (placebo-subtracted treatment difference of 3.43 [2.17, 4.70] h, *p* < 0.001). Importantly, OFF time reductions were apparent even though over 70% of patients were already on adjunctive medications, including >50% on a dopamine agonist, >35% on an MAO-B inhibitor, and >35% on a COMT inhibitor. Reductions in OFF time were accompanied by statistically significant and clinically meaningful reductions in MDS-UPDRS Part II motor experiences of daily living^21^ (driven by significant improvements in freezing, tremor, getting out of bed/deep chair, turning in bed, eating tasks, and speech) and reductions in MDS-UPDRS Part IV motor fluctuation subscales^22^ (including significant improvement in time spent in the OFF state, impact of fluctuations, and painful OFF-state dystonia).

Our results also indicate that even patients with <2.5 h OFF time at baseline experience a significant (albeit smaller) treatment effect of amantadine-DR/ER on OFF time (Week 12 treatment difference of −0.77 [−1.49, −0.06] h versus placebo). In this subgroup, the observation that daily OFF time was maintained at baseline levels for amantadine-DR/ER-treated patients compared to a progressive increase in placebo-treated patients, could potentially suggest a delay or reduction in the emergence of motor fluctuations. Alternatively, individual patients may exhibit variable degrees of motor complications each day, and benefit may be reflected in keeping patients with low levels of reported fluctuations at or near these low levels. Indeed, responder analyses confirmed that reductions in OFF time were greater for amantadine DR/ER versus placebo across participant groups with varying levels of baseline OFF time.

Our findings also demonstrate that the therapeutic response was maintained for as long as two years. This is of practical importance to highlight, because the sustainability of the antidyskinetic response to immediate-release amantadine was debated at the turn of the century^23,24^, despite some evidence of long-term efficacy^25^. Lingering concerns in the field about tachyphylaxis may have resulted in the underuse of this important treatment option^26,27^. The persistence of benefit of amantadine-DR/ER over several years in reducing dyskinesias and motor fluctuations is therefore notable.

Our results also highlight that most patients recruited to dyskinesia trials (88% in the pooled analysis) also experience OFF motor fluctuations at baseline. This finding is consistent with prevalence studies suggesting a majority of patients with dyskinesia also have to deal with OFF episodes^1,2,28,29^. Accumulated information indicates that amantadine DR/ER reduces dyskinesia and OFF time, and improves motor aspects of experiences of daily living^8–11^^,14^. Moreover, prior amantadine DR/ER diary analyses show significantly reduced numbers of transitions between OFF and dyskinesia states with a consolidation of periods of ‘good’ ON time (without troublesome dyskinesia), to the extent that the length of the first episode of the day of ‘good’ ON time increased by a mean of 5.2 h (vs. 2 h for placebo)^30^.

Although the potential benefits of amantadine in reducing both OFF time and dyskinesia were previously noted in a small study of 18 patients with advanced PD by Metman et al^31^, hitherto this benefit has not been replicated in other trials of amantadine. One possible reason for this is the omission of motor fluctuation outcomes in most amantadine trials. However, it should also be recognized that because amantadine-DR/ER contains a higher amantadine dose than typically used in immediate-release amantadine regimens, is given at nighttime to provide high plasma levels early in the morning, and because it provides sustained plasma concentrations throughout the waking day^32^, the results of this trial are not broadly generalizable to other amantadine products. In addition, the mechanisms underlying amantadine-DR/ER’s ability to reduce both dyskinesia and OFF time are not precisely known. This ‘dual’ therapeutic effect may potentially be explained by amantadine’s multi-modal action; while the anti-glutamatergic (NMDA) effects of amantadine are now regarded as the central mechanism of its antidyskinetic action^33^, its effects on dopamine transmission could explain some or all of its antiparkinsonian efficacy^34^. Moreover, the long term pulsatile stimulation of striatal dopamine receptors due to the intermittent delivery of levodopa-derived dopamine may induce changes in synaptic plasticity as well as cause dysregulation in the natural diurnal modulation of both glutamatergic and dopaminergic tone, whereas high and consistent early morning levels of amantadine may exert a positive effect^35^. It is interesting to note that in our analysis there was no apparent correlation between the amantadine-DR/ER-related reductions in OFF time and reductions in troublesome dyskinesia, potentially suggesting these benefits are mediated by separate mechanisms.

Strengths of the present analyses lie in the similar design and conduct of the two pivotal trials which allowed pooling of the data and enable robust analysis of subgroups. Our main analyses are derived from double-blind trials. Since the trials were focused on dyskinesia reduction, the risk of introducing expectation bias to patient-reported assessments of the secondary measure of OFF time is likely to have been minimized. In addition, to our knowledge, the EASE LID 2 trial is the longest-running trial to evaluate amantadine in PD patients with motor complications. Limitations include the smaller size and *post-hoc* nature of the subgroup analyses (although motor states were prospectively assessed), the open-label nature of the follow-on trial, the lack of a quality-of-life measure, and the fact that patients were recruited based on dyskinesia. While the cut-off of 2.5 h for subgroup analyses was derived based on two rationales (i.e. median OFF time in the mITT population as well as being in line with the motor fluctuation trials in the literature), the mean OFF time in the ≥2.5 h group of 4.4 h is still somewhat less than the >5–6 h observed in clinical trials of oral interventions aimed at reducing motor fluctuations^30,36–38^. Other studies have shown that patients with greater OFF time at baseline are likely to experience the largest absolute reduction in hours OFF^39,40^.

Current treatment algorithms often present a difficult trade-off between managing OFF time and managing dyskinesia. The results of these pooled analyses show a robust effect of amantadine-DR/ER in reducing OFF time in patients experiencing dyskinesia and at least 2.5 h of OFF time at baseline. Patients with less OFF time also experienced benefit with amantadine-DR/ER compared to treatment with placebo. The benefits in reducing OFF time and the disability caused by motor fluctuations persisted for as long as 2 years with open-label treatment. Amantadine-DR/ER (ADS 5102, Gocovri^®^) is now approved in the US as an adjunct to levodopa/carbidopa in PD patients with OFF episodes and/or dyskinesia^15^.

## Methods

### Trial designs and participants

The efficacy and safety of amantadine-DR/ER as an antidyskinetic agent in PD was established in two phase III pivotal trials^9,10^, followed by an open-label extension trial^41^. The full methodologic details of these trials have been previously published:

1. EASE LID: A randomized, double-blind, placebo-controlled, 25-week clinical trial (NCT02136914)^9^.
2. EASE LID 3: A randomized, double-blind, placebo-controlled, 12-week trial (NCT02274766)^10^.
3. EASE LID 2: An open-label 2-year trial (NCT02202551), including participants from EASE-LID and EASE LID 3^41^.

Briefly, participants (aged 30–85 years old) in EASE LID and EASE LID 3 were required to be experiencing ≥1 h/day (2 half-h intervals) of ON time with troublesome dyskinesia between 9 am and 4 pm, on the two days preceding treatment initiation, as documented by entries in PD home diaries. Dyskinesia was required to be causing at least mild functional impairment, as documented at screening and baseline by a score ≥2 on item 4.2 of the MDS-UPDRS. Enrolled participants were randomized in a 1: 1 ratio to double-blind amantadine-DR/ER or placebo once daily at bedtime. Amantadine-DR/ER was initiated at 137 mg/day (corresponding to 170 mg of amantadine HCl) for the first week, and titrated to 274 mg/day (corresponding to 340 mg of amantadine HCl) thereafter. Levodopa preparations, which had to be administered ≥3 times daily for eligibility, and all other antiparkinsonian medications were to be unchanged for ≥30 days prior to screening and during trial participation.

Participants completing these double-blind trials could continue into EASE LID 2 and receive open-label amantadine-DR/ER for up to 101 weeks, with or without a gap between double-blind and open-label trials^41^. Participants previously excluded from the pivotal trials due to the use of a deep brain stimulation device and those who wished to enroll after completing an earlier phase II trial were also eligible but are not included in the present analyses. As in the pivotal trials, all participants were initiated at an amantadine-DR/ER dose of 137 mg/day for the first open-label trial week and titrated to 274 mg/day starting Week 2. Participants were allowed to change their PD medications (including levodopa dosage) as needed during the open-label trial, and the amantadine-DR/ER dose was tapered back to 137 mg for the final week (week 101)^41^.

### Ethics

All three trials were conducted in compliance with the Declaration of Helsinki and International Conference on Harmonization (ICH) Good Clinical Practice (GCP) guidelines. The protocol and study procedures were approved by the following Institutional review boards (IRB) and ethical committees: Baylor College of Medicine IRB, Beth Israel Medical Center IRB, Biomedical Research Alliance of New York LLC IRB, CEIC del Hospital de la Santa Creu I Sant Pau, Chesapeake Institutional Review Board, Cleveland Clinic IRB, Comite de Protection des Personnes Sud Ouest et Outre Mer ll, Copernicus Group IRB, Ethics Committee of the Innsbruck Medical University, Ethik-Kommission des Fachbereichs Medizin der Phillips-Universtat Marburg, Henry Ford Health System IRB, Johns Hopkins University IRB, Mayo Clinic IRB, Park Nicollet Institute IRB, Penn State College of Medicine IRB, Rush University Medical Center IRB, St. Joseph’s Hospital and Medical Center IRB for Human Research, UC Davis Medical Center IRB, University Health Network Ethics Research Board, University of Kansas Medical Center Human Subjects Committee, University of Miami, Human Subjects Research Office, University of Pennsylvania IRB, University of Saskatchewan Biomedical Research Ethics Board, UT Southwestern IRB, Washington University School of Medicine IRB, and Western IRB. All participants provided written informed consent before any procedures were performed.

### Efficacy outcomes

The primary efficacy outcome measure in the double-blind trials was changed from baseline in Unified Dyskinesia Rating Scale (UDysRS) total score, as assessed at week 12^9,10^. All clinic-based study assessments were to be conducted when participants were in the ON state and experiencing their typical dyskinesia. Relevant to the present analyses, as a key secondary outcome, all participants completed home diaries for the two consecutive days prior to each scheduled visit where they categorized their predominant motor state during each half-hour interval of the 24-h day as OFF, ON without dyskinesia, ON with non-troublesome dyskinesia, ON with troublesome dyskinesia, or asleep^42^. Participants and care partners received training on how to use the diaries, and concordance with the diary was confirmed during the screening period. Diaries with ≥4 missing entries (i.e., missing 2 h) per day were considered unevaluable for analysis. Otherwise, missing data were imputed by assigning the 30 minutes of each missing interval, in equal portions of 15 min each, to the responses of the immediately preceding and subsequent completed (non-missing) intervals.

The MDS-UPDRS and Clinician Global Impression of Change (CGI-C), were also completed at baseline and at weeks 2, 8, and 12 of both double-blind trials. The MDS-UPDRS was the only PD rating instrument used in the open-label trial, with MDS-UPDRS Part IV (items 4.1–4.6) being the principal assessment of motor complications. For the present analyses we separately evaluated Part IV motor fluctuation (items 4.3–4.6) and dyskinesia (4.1–4.2) subscores in the pooled double-blind analyses, and also evaluated Part IV motor fluctuation subscores through the open-label study to specifically look for the persistence of OFF time reduction through 100 weeks of treatment.

### Analyses

Analyses were performed for

1. The modified intent-to-treat (mITT) population, comprising all randomized participants who were exposed to the study drug and provided ≥1 post-baseline assessment.
2. Participants with at least 2.5 h of OFF time at baseline (≥2.5 h subgroup). This is consistent with inclusion criteria for many previous trials of other interventions aimed at reducing motor fluctuations^38,43,44^ and was also the median amount of OFF time in the mITT population.
3. Participants with less than 2.5 h of OFF time at baseline (<2.5 h subgroup), including those with zero hours OFF to ensure capture of any OFF-time emergence.

Except for the MDS-UPDRS item scores and subgroupings, all mITT population outcomes shown here were pre-specified analyses in the statistical plan for data pooling and were evaluated using mixed effect model repeat measurement (MMRM) analyses, or in the case of CGI-C, the Cochrane–Mantel–Haenszel test. All analyses were set at a two-sided, 5% significance level and were performed using SAS version 9.4 (SAS Institute Inc., Cary, North Carolina). Subgroup analyses were conducted *post hoc*, using the same analytical methods as for the mITT population. Correlation-regression analyses were also performed *post hoc* to test for potential associations between (baseline to Week 12) changes in OFF time and changes in ON time with troublesome dyskinesia for the mITT population. In addition, we conducted a Week 12 responder analysis of reductions in OFF time by standard percentage thresholds of not improved/worse, ≥25% reduction, ≥50% reduction, ≥75% reduction, and 100% reduction. Open label data are reported descriptively.

### Reporting summary

Further information on research design is available in the Nature Research Reporting Summary linked to this article.

## Supplementary information

Supplemental Appendix

Reporting Summary

## Data Availability

Access to datasets generated or analyzed for this publication can be made available to independent researchers following receipt of a research proposal, data analysis plan, and summary of researcher qualifications. Requests may be submitted to Supernus Pharmaceuticals at MedAffairs@supernus.com. Provision of data is contingent on business feasibility and execution of a data use agreement.
