# Supplementary material for: Amantadine delayed release/extended release capsules significantly reduce OFF time in Parkinson’s disease
Source: NPJ Parkinsons Dis. 2022 Mar 18;8:29. doi: 10.1038/s41531-022-00291-1 (PMC8933492; doi:10.1038/s41531-022-00291-1)
Supplement: Supplementary file 1 — Supplemental Appendix [file 41531_2022_291_MOESM1_ESM.pdf]

Supplemental Appendix

Supplemental Figure e1. Scatter Plot with regression line and analysis of change from baseline in ON time with troublesome dyskinesia versus change from baseline in OFF time at Week 12 (mITT population). For each treatment, quadrants are defined based on the respective sample means for that treatment group.

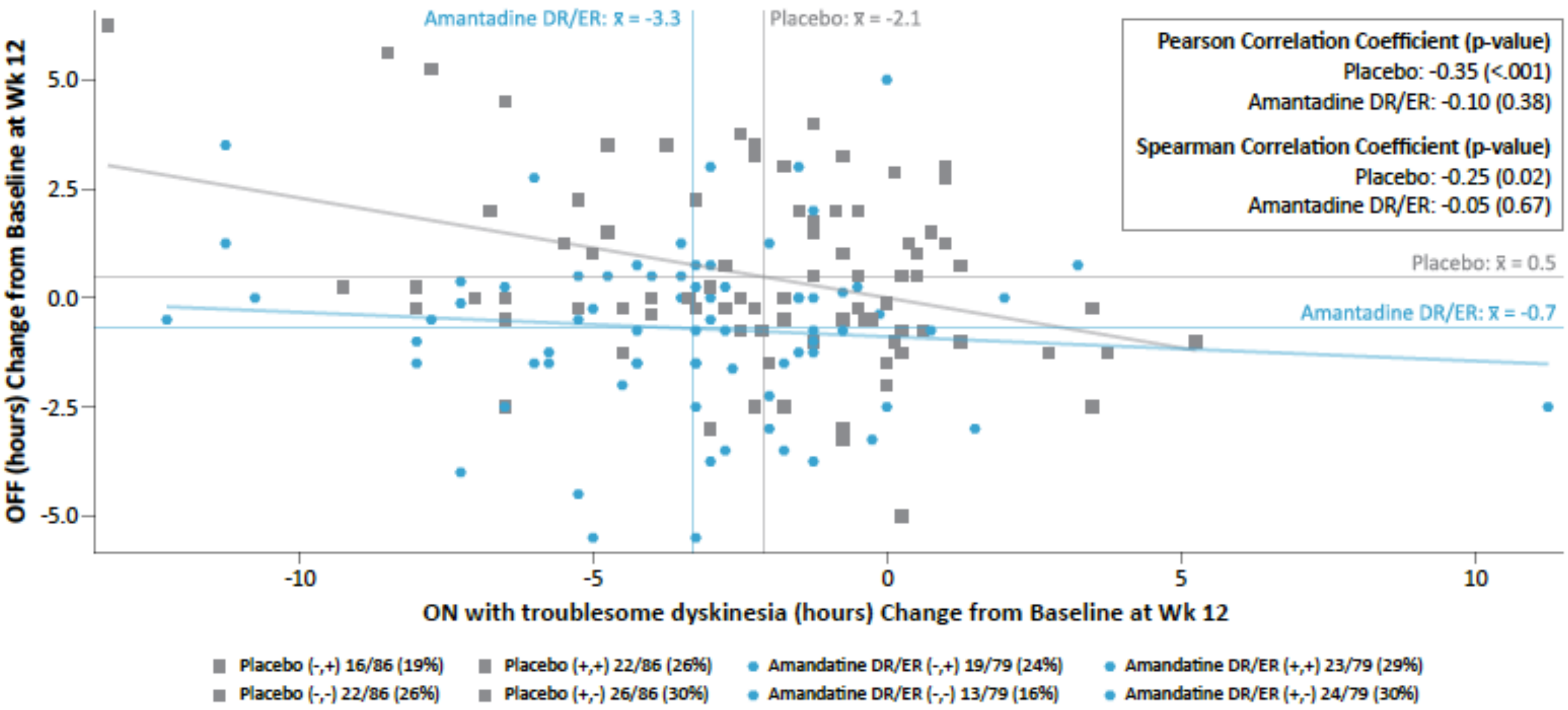

Supplemental Figure e2. Least squares mean [amantadine DR/ER – placebo] treatment differences in MDS-UPDRS Part I and II scores for patients with ≥2.5 hours OFF at Baseline in pooled pivotal trials (a) Part I (non-motor experiences of daily living), (b) Part II (motor experiences of daily living).

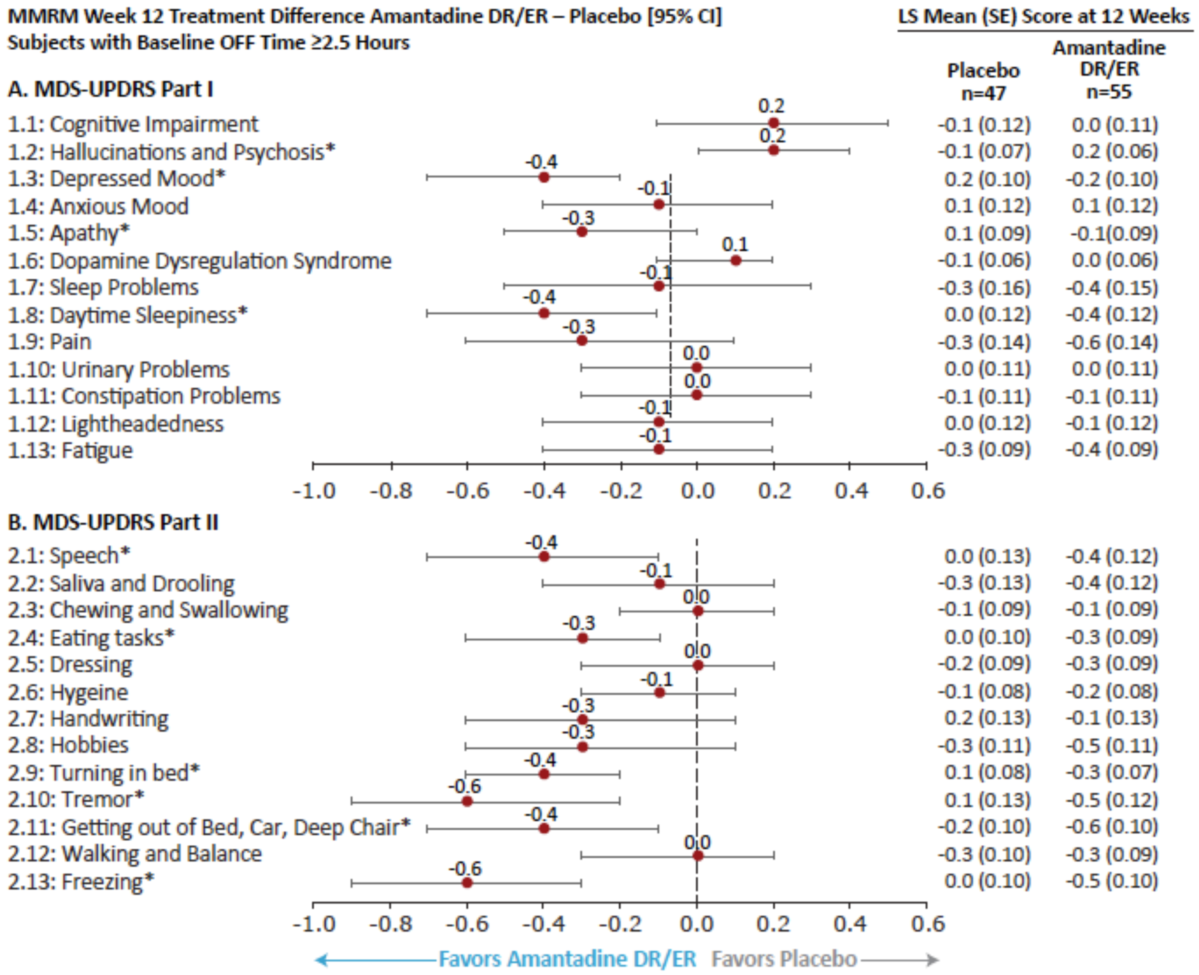

Supplemental e1. Week 12 responder analysis of reductions in OFF time by standard percentage thresholds of not improved/worse, ≥25% reduction, ≥50% reduction, ≥75% reduction, and 100% reduction.

OFF time, percent reduction, %	All Subjects		Baseline OFF ≥2.5 hours		Baseline OFF <2.5 hours	
	Placebo (n=86)	Amantadine DR/ER (n=79)	Placebo (n=42)	Amantadine-DR/ER (N=46)	Placebo (n=86)	Amantadine DR/ER (n=79)
**Not improved/worse[a]**	50%	32%	48%	30%	52%	33%
**Subjects with baseline OFF >0[b]**	**(n=76)**	**(n=71)**	**(n=42)**	**(n=46)**	**(n=34)**	**(n=25)**
**Any OFF time reduction**	51%	66%	52%	70%	50%	60%
**≥25% reduction**	37%	54%	33%	52%	41%	56%
**≥50% reduction**	22%	32%	19%	26%	27%	44%
**≥75% reduction**	15%	21%	10%	15%	21%	32%
**100% reduction**	11%	18%	5%	11%	18%	32%

[a]*Patients with no OFF time at baseline are only counted as "not improved/worse" if they subsequently report OFF time at Week 12.*
[b]*Only patients with OFF time at baseline (and therefore with potential to show improvement) are included in the responder analysis of standard percentage thresholds. Sample sizes in this row show the number of subjects in each group with OFF time at baseline (thereby providing the denominators for the responder analysis). DR, delayed release; ER, extended release.*

Supplemental Table e2. LS Mean (SE) changes from baseline in MDS-UPDRS Part IV subscores and item scores by visit, mITT population and by baseline OFF time

	mITT (n=196)			Baseline OFF ≥2.5 hours (N=102)			Baseline OFF <2.5 hours (N=94)		
	Placebo (N=96)	Amantadine-DR/ER (N=100)	Mean [95%CI] Treatment difference	Placebo (N=47)	Amantadine-DR/ER (N=55)	Mean [95%CI] Treatment difference	Placebo (N=49)	Amantadine-DR/ER (N=45)	Mean [95%CI] Treatment difference
**MDS-UPDRS Part IV change from baseline; LS mean ± SE**									
Week 2	-1.3 ± 0.3	-4.0 ± 0.3	-2.7 [-3.6, -1.9]	-1.3 ± 0.5	-3.7 ± 0.4	-2.5 [-3.7, -1.2]	-1.2 ± 0.4	-4.2 ± 0.4	-3.0 [-4.2, -1.9]
Week 8	-2.3 ± 0.3	-4.2 ± 0.3	-1.9 [-2.8, -1.0]	-2.1 ± 0.5	-3.8 ± 0.4	-1.7 [-2.9, -0.5]	-2.3 ± 0.3	-4.2 ± 0.3	-1.9 [-2.8, -1.0]
Week 12	-2.1 ± 0.3	-4.4 ± 0.3	- 2.3 [-3.2, -1.5]	-1.5 ± 0.4	-4.2 ± 0.4	-2.7 [-3.8, -1.6]	-2.1 ± 0.3	-4.4 ± 0.3	-2.3 [-3.2, -1.5]
**Dyskinesia items total subscore (4.1+4.2) change from baseline; LS mean ± SE**									
Week 2	-0.7 ± 0.2	-2.3 ± 0.2	-1.6 [-2.1, -1.2]	-0.8 ± 0.2	-2.3 ± 0.2	-1.5 [-2.2, -0.9]	-0.7 ± 0.2	-2.4 ± 0.2	-1.8 [-2.4, -1.1]
Week 8	-1.4 ± 0.2	-2.4 ± 0.2	-1.0 [-1.5, -0.5]	-1.2 ± 0.2	-2.4 ± 0.2	-1.2 [-1.9, -0.6]	-1.7 ± 0.2	-2.5 ± 0.3	-0.8 [-1.5, -0.0]
Week 12	-1.3 ± 0.2	-2.6 ± 0.2	-1.2 [-1.7, -0.7]	-1.1 ± 0.2	-2.3 ± 0.2	-1.2 [-1.9, -0.6]	-1.6 ± 0.3	-2.9 ± 0.3	-1.3 [-2.0, -0.5]
**Motor fluctuations items total subscore (4.3+4.4+4.5+4.6) change from baseline; LS mean ± SE**									
Week 2	-0.6 ± 0.2	-1.6 ± 0.2	-1.1 [-1.7, -0.5	-0.5 ± 0.3	-1.5 ± 0.29	-1.0 [-1.8, -0.2]	-0.6 ± 0.32	-1.8 ± 0.34	-1.3 [-2.2, -0.4]
Week 8	-0.9 ± 0.3	-1.7 ± 0.3	-0.9 [-1.6, -0.2]	-0.9 ± 0.3	-1.4 ± 0.30	-0.5 [-1.4, 0.3]	-0.7 ± 0.37	-2.2 ± 0.41	-1.5 [-2.6, -0.4]
Week 12	-0.7 ± 0.2	-1.8 ± 0.2	-1.1 [-1.7, -0.4]	-0.3 ± 0.3	-1.8 ± 0.26	-1.5 [-2.2, -0.8]	-1.0 ± 0.37	-1.8 ± 041	-0.8 [-1.9, 0.3]
**Item 4.1 (time spent with dyskinesia) change from baseline; LS mean ± SE**									
Week 2	-0.2 ± 0.1	-0.9 ± 0.1	-0.7 [-1.0, -0.4]	-0.1 ± 0.2	-0.8 ± 0.1	-0.6 [-1.0, -0.2]	-0.2 ± 0.1	-1.1 ± 0.1	-0.8 [-1.2, -0.4]
Week 8	-0.5 ± 0.1	-0.9 ± 0.1	-0.4 [-0.6, -0.1]	-0.4 ± 0.1	-0.8 ± 0.1	-0.3 [-0.7, 0.0]	-0.6 ± 0.2	-1.0 ± 0.2	-0.4 [-0.9, 0.0]
Week 12	-0.6 ± 0.1	-1.0 ± 0.1	-0.5 [-0.8, -0.2]	-0.4 ± 0.1	-0.8 ± 0.1	-0.4 [-0.8, -0.1]	-0.7 ± 0.2	-1.3 ± 0.2	-0.6 [-1.0, -0.1]
**Item 4.2 (functional impact of dyskinesia) change from baseline; LS mean ± SE**									
Week 2	-0.5 ± 0.1	-1.5 ± 0.1	-1.0 [-1.2, -0.7]	-0.6 ± 0.2	-1.5 ± 0.1	-0.9 [-1.3, -0.5]	-0.4 ± 0.1	-1.4 ± 0.1	-1.0 [-1.3, -0.6]
Week 8	-0.9 ± 0.1	-1.6 ± 0.1	-0.6 [-0.9, -0.4]	-0.7 ± 0.1	-1.7 ± 0.1	-0.9 [-1.3, -0.5]	-1.1 ± 0.1	-1.5 ± 0.2	-0.4 [-0.8, 0.1]
Week 12	-0.8 ± 0.1	-1.5 ± 0.1	-0.8 [-1.0, -0.6]	-0.7 ± 0.2	-1.5 ± 0.1	-0.8 [-1.2, -0.4]	-0.9 ± 0.1	-1.6 ± 0.2	-0.7 [-1.2, -0.3]

**Item 4.3 (time spent in the OFF state) change from baseline; LS mean ± SE**									
Week 2	-0.0 ± 0.1	-0.2 ± 0.1	-0.2 [-0.3, -0.0]	0.0 ± 0.1	-0.2 ± 0.1	-0.3 [-0.5, -0.1]	-0.0 ± 0.1	-0.1 ± 0.1	-0.1 [-0.3, 0.1]
Week 8	0.0 ± 0.1	-0.2 ± 0.1	-0.3 [-0.5, -0.1]	0.0 ± 0.1	-0.2 ± 0.1	-0.3 [-0.5, -0.0]	0.1 ± 0.1	-0.3 ± 0.1	-0.3 [-0.6, -0.1]
Week 12	0.0 ± 0.1	-0.3 ± 0.1	-0.3 [-0.5, -0.2]	0.1 ± 0.1	-0.3 ± 0.1	-0.4 [-0.6, -0.2]	0.0± 0.1	-0.3 ± 0.1	-0.3 [-0.5, -0.1]
**Item 4.4 (functional impact of fluctuations) change from baseline; LS mean ± SE**									
Week 2	-0.3 ± 0.1	-0.8 ± 0.1	-0.5 [-0.8, -0.2]	-0.1 ± 0.2	-0.8 ± 0.2	-0.6 [-1.1, -0.2]	-0.4 ± 0.2	-0.8 ± 0.2	-0.5 [-0.9, -0.0]
Week 8	-0.6 ± 0.1	-0.8 ± 0.1	-0.3 [-0.6, 0.1]	-0.4 ± 0.2	-0.8 ± 0.2	-0.4 [-0.9, 0.0]	-0.7 ± 0.2	-0.9 ± 0.2	-0.2 [-0.7, 0.3]
Week 12	-0.4 ± 0.1	-0.8 ± 0.1	-0.4 [-0.7, -0.1]	-0.3 ± 0.2	-0.9 ± 0.2	-0.6 [-1.0, -0.2]	-0.6 ± 0.2	-0.8 ± 0.2	-0.3 [-0.8, 0.2]
**Item 4.5 (complexity of motor fluctuations) change from baseline; LS mean ± SE**									
Week 2	-0.1 ± 0.1	-0.4 ± 0.10	-0.3 [-0.6, -0.0]	-0.2 ± 0.14	-0.3 ± 0.13	-0.1 [-0.5, 0.3]	-0.0 ± 0.1	-0.5 ± 0.2	-0.5 [-0.9, -0.1]
Week 8	-0.2 ± 0.1	-0.3 ± 0.10	-0.2 [-0.4, 0.1]	-0.2 ± 0.13	-0.1 ± 0.12	0.0 [-0.3, 0.4]	-0.1 ± 0.1	-0.6 ± 0.2	-0.5 [-0.9, -0.0]
Week 12	-0.2 ± 0.1	-0.3 ± 0.10	-0.1 [-0.3, 0.2]	-0.1 ± 0.12	-0.2 ± 0.12	-0.1 [-0.4, 0.2]	-0.3 ± 0.2	-0.3 ± 0.2	-0.0 [-0.5, 0.4]
**Item 4.6 (painful OFF-state dystonia) change from baseline; LS mean ± SE**									
Week 2	-0.2 ± 0.1	-0.3 ± 0.10	-0.1 [-0.4, 0.1]	-0.3 ± 0.14	-0.3 ± 0.13	0.0 [-0.4, 0.4]	-0.1 ± 0.1	-0.4 ± 0.1	-0.3 [-0.6, 0.1]
Week 8	-0.2 ± 0.1	-0.3 ± 0.11	-0.2 [-0.5, 0.1]	-0.4 ± 0.15	-0.2 ± 0.14	0.1 [-0.3, 0.5]	0.0 ± 0.2	-0.5 ± 0.2	-0.5 [-1.0, -0.0]
Week 12	-0.1 ± 0.1	-0.4 ± 0.10	-0.3 [-0.6, 0.0]	-0.1 ± 0.15	-0.5 ± 0.14	-0.4 [-0.8, -0.0]	-0.2 ± 0.1	-0.5 ± 0.2	-0.3 [-0.7, 0.1]

Supplemental Table e3. Change from double-blind baseline in MDS-UPDRS Part 4 motor fluctuation subscores at baseline, and weeks 8, 52, 100 (endpoint) and last visit (LOCF) of the open-label trial for patients continuing into the open-label extension

Change from double-blind baseline	Placebo then amantadine- DR/ER (N=81)	Amantadine- DR/ER (N=64)	Placebo then Amantadine-DR/ER ≥2.5hr OFF at Baseline (N=40)	Amantadine- DR/ER ≥2.5hr OFF at Baseline (N=35)	Placebo then Amantadine- DR/ER <2.5hr OFF at Baseline (N=41)	Amantadine- DR/ER <2.5hr OFF at Baseline (N=29)
**Motor fluctuations Part IV**						
Baseline open-label	-1.8 (2.9)	-4.4 (3.8)	-1.1 (3.0)	-3.8 (34)	-2.5 (2.8)	-4.7 (2.2)
Week 8 open-label	-4.8 (3.2)	-4.8 (3.7)	-4.9 (4.0)	-4.9 (3.2)	-4.7 (2.2)	-4.7 (4.3)
Week 52 open-label	-4.3 (3.3)	-4.7 (3.6)	-4.0 (3.8)	-5.0 (2.9)	-4.8 (2.6)	-4.3 (4.3)
Week 100 open-label	-4.0 (3.5)	-4.0 (3.2)	-4.2 (3.9)	-3.8 (3.4)	-3.7 (3.0)	-4.4 (2.7)
Last on-study measurement	-3.8 (3.4)	-3.9 (4.3)	-3.8 (3.9)	-4.1 (3.7)	-3.8 (3.0)	-3.6 (5.0)
**Motor fluctuations subscore (4.3-4.6)**						
Baseline open-label	-0.5 (2.5)	-1.9 (2.7)	-0.1 (2.2)	-1.4 (2.0)	-0.9 (2.7)	-2.6 (3.3)
Week 8 open-label	-2.0 (2.4)	-1.7 (2.8)	-2.2 (2.8)	-1.7 (2.0)	-1.7 (1.9)	-1.7 (3.6)
Week 52 open-label	-1.4 (2.6)	-1.7 (3.1)	-1.3 (2.8)	-2.1 (2.2)	-1.5 (2.4)	-1.3 (3.9)
Week 100 open-label	-1.3 (2.8)	-1.3 (2.7)	-1.8 (3.0)	-1.3 (2.5)	-0.6 (2.9)	-1.4 (3.0)
Last on-study measurement	-1.3 (2.8)	-1.4 (3.2)	-1.4 (3.0)	-1.5 (2.6)	-1.1 (2.7)	-1.2 (3.8)
**4.3: Time spent OFF**						
Baseline open-label	0.0 (0.6)	-0.3 (0.6)	0.0 (0.6)	0.2 (0.6)	0.0 (0.6)	-0.3 (0.7)
Week 8 open-label	-0.3 (0.7)	-0.2 (0.6)	-0.4 (0.8)	-0.2 (0.6)	-0.2 (0.6)	-0.1 (0.6)
Week 52 open-label	-0.2 (0.7)	-0.3 (0.6)	-0.3 (0.7)	-0.3 (0.7)	-0.2 (0.7)	-0.3 (0.6)
Week 100 open-label	-0.3 (0.7)	-0.1 (0.7)	-0.3 (0.8)	-0.2 (0.7)	-0.2 (0.5)	-0.1 (0.8)
Last on-study measurement	-0.3 (0.8)	-0.2 (0.8)	-0.4 (1.0)	-0.1 (0.8)	-0.1 (0.7)	-0.2 (0.7)

**4.4: Functional impact of fluctuations**						
Baseline open-label	-0.4 (1.3)	-0.9 (1.3)	-0.2 (1.3)	-0.8 (1.0)	-0.6 (1.3)	-1.0 (1.5)
Week 8 open-label	-0.9 (1.3)	-0.8 (1.3)	-1.1 (1.4)	-1.1 (1.2)	-0.8 (1.1)	-0.6 (1.4)
Week 52 open-label	-0.7 (1.2)	-0.9 (1.5)	-0.6 (1.3)	-1.3 (1.4)	-0.8 (1.1)	-0.6 (1.3)
Week 100 open-label	-0.6 (1.4)	-0.8 (1.4)	-0.8 (1.3)	-1.0 (1.4)	-0.4 (1.4)	-0.5 (1.5)
Last on-study measurement	-0.6 (1.4)	-0.6 (1.5)	-0.8 (1.3)	-0.8 (1.4)	-0.4 (1.4)	-0.3 (1.7)
**4.5: Complexity of fluctuations**						
Baseline open-label	-0.1 (1.1)	-0.3 (1.1)	0.0 (0.9)	0.0 (1.0)	-0.3 (1.3)	-0.6 (1.2)
Week 8 open-label	-0.3 (1.1)	0.0 (1.5)	-0.3 (1.0)	0.2 (1.4)	-0.3 (1.3)	-0.2 (1.5)
Week 52 open-label	-0.3 (1.0)	-0.3 (1.3)	-0.2 (1.0)	-0.3 (1.4)	-0.3 (1.1)	-0.3 (1.3)
Week 100 open-label	-0.2 (1.0)	0.2 (1.1)	-0.5 (0.9)	0.3 (1.2)	0.0 (1.1)	-0.1 (1.0)
Last on-study measurement	-0.2 (1.3)	-0.1 (1.3)	-0.2 (1.3)	0.1 (1.3)	-0.3 (1.3)	-0.2 (1.2)
**4.6: OFF state dystonia**						
Baseline open-label	0.0 (1.1)	-0.5 (1.5)	0.1 (1.2)	-0.4 (1.5)	-0.0 (0.9)	-0.7 (1.5)
Week 8 open-label	-0.4 (1.1)	-0.7 (1.4)	-0.4 (1.3)	-0.6 (1.1)	-0.4 (0.9)	-0.8 (1.6)
Week 52 open-label	-0.2 (1.2)	-0.3 (1.6)	-0.1 (1.3)	-0.3 (1.2)	-0.3 (1.2)	-0.4 (1.9)
Week 100 open-label	-0.1 (1.4)	-0.6 (1.4)	-0.2 (1.5)	-0.5 (1.1)	0.0 (1.3)	-0.7 (1.8)
Last on-study measurement	-0.2 (1.2)	-0.6 (1.5)	-0.1 (1.2)	-0.6 (1.1)	-0.3 (1.1)	-0.5 (1.8)
**Dyskinesia Subscore**						
Baseline open-label	-1.3 (1.6)	-2.5 (1.9)	-1.0 (1.6)	-2.4 (2.0)	-1.6 (1.59)	-2.6 (1.86)
Week 8 open-label	-2.8 (1.6)	-3.1 (1.6)	-2.7 (1.8)	-3.2 (1.7)	-3.0 (1.39)	-2.9 (1.46)
Week 52 open-label	-3.0 (1.6)	-2.9 (1.7)	-2.7 (1.7)	-2.9 (1.7)	-3.3 (1.43)	-3.0 (1.72)
Week 100 open-label	-2.7 (1.6)	-2.6 (1.8)	-2.4 (1.4)	-2.5 (2.0)	-3.1 (1.64)	-3.0 (1.35)
Last on-study measurement	-2.6 (1.8)	-2.5 (2.0)	-2.4 (1.6)	-2.5 (2.1)	-2.7 (1.9)	-2.4 (1.9)

Samples Sizes at time points following open-label baseline for the group that received placebo during double-blind were (mitt, ≥2.5 h, <2.5 h, respectively): Open-label week 8 (n=75, n=38, n=37), week 52 (57, 31, 26), Week 100 (44, 23, 21), Last On Study Visit (71, 37, 34). Samples Sizes at time points following open-label baseline for the group that received amantadine DR/ER during double-blind were (mitt, ≥2.5 h, <2.5 h, respectively): Open-label week 8 (n=64, n=35, n=29), week 52 (51, 28, 23), week 100 (39, 26, 13), Last On Study Visit (56 , 30, 26)
