# Supplementary material for: Amantadine delayed release/extended release capsules significantly reduce OFF time in Parkinson’s disease
Source: NPJ Parkinsons Dis. 2022 Mar 18;8:29. doi: 10.1038/s41531-022-00291-1 (PMC8933492; doi:10.1038/s41531-022-00291-1)
Supplement: Supplementary file 2 — Reporting Summary [file 41531_2022_291_MOESM2_ESM.pdf]

nature portfolio

Corresponding author(s): DBPR NPJPARKD-00791R

Last updated by author(s): Oct 12, 2021

Reporting Summary

Nature Portfolio wishes to improve the reproducibility of the work that we publish. This form provides structure for consistency and transparency in reporting. For further information on Nature Portfolio policies, see our Editorial Policies and the Editorial Policy Checklist.

Statistics

For all statistical analyses, confirm that the following items are present in the figure legend, table legend, main text, or Methods section.

n/a	Confirmed	
☐	☒	The exact sample size (n) for each experimental group/condition, given as a discrete number and unit of measurement
☐	☒	A statement on whether measurements were taken from distinct samples or whether the same sample was measured repeatedly
☐	☒	The statistical test(s) used AND whether they are one- or two-sided<br>*Only common tests should be described solely by name; describe more complex techniques in the Methods section.*
☐	☒	A description of all covariates tested
☐	☒	A description of any assumptions or corrections, such as tests of normality and adjustment for multiple comparisons
☐	☒	A full description of the statistical parameters including central tendency (e.g. means) or other basic estimates (e.g. regression coefficient) AND variation (e.g. standard deviation) or associated estimates of uncertainty (e.g. confidence intervals)
☐	☒	For null hypothesis testing, the test statistic (e.g. F, t, r) with confidence intervals, effect sizes, degrees of freedom and P value noted<br>*Give P values as exact values whenever suitable.*
☒	☐	For Bayesian analysis, information on the choice of priors and Markov chain Monte Carlo settings
☒	☐	For hierarchical and complex designs, identification of the appropriate level for tests and full reporting of outcomes
☐	☒	Estimates of effect sizes (e.g. Cohen's d, Pearson's r), indicating how they were calculated

Our web collection on statistics for biologists contains articles on many of the points above.

Software and code

Policy information about availability of computer code

Data collection	The software used for data collection was Medidata Rave, version 5.6.4.52, Medidata Solutions, Inc. www.mdsol.com
Data analysis	SAS version 9.4 (SAS Institute Inc., Cary, North Carolina)

For manuscripts utilizing custom algorithms or software that are central to the research but not yet described in published literature, software must be made available to editors and reviewers. We strongly encourage code deposition in a community repository (e.g. GitHub). See the Nature Portfolio guidelines for submitting code & software for further information.

Data

Policy information about availability of data

All manuscripts must include a data availability statement. This statement should provide the following information, where applicable:

- Accession codes, unique identifiers, or web links for publicly available datasets
- A description of any restrictions on data availability
- For clinical datasets or third party data, please ensure that the statement adheres to our policy

The datasets generated during and/or analyzed during the current study are available from Adamas Pharmaceuticals on reasonable request. Adamas Pharmaceuticals Inc will share study documents with qualified researchers who provide valid research questions. Requests should be directed to info@adamaspharma.com

Field-specific reporting

Please select the one below that is the best fit for your research. If you are not sure, read the appropriate sections before making your selection.

☒ Life sciences ☐ Behavioural & social sciences ☐ Ecological, evolutionary & environmental sciences

For a reference copy of the document with all sections, see nature.com/documents/nr-reporting-summary-flat.pdf

Life sciences study design

All studies must disclose on these points even when the disclosure is negative.

Sample size	Pooled analysis of previously published Phase 3 trials
Data exclusions	No exclusions, except for the responder analyses in which participants who had 0 hours of OFF at baseline were excluded because there was no room for improvement (as noted in paper).
Replication	Reproducibility is shown between EASE LID and EASE LID 3 which had similar results separately. This paper is a report of the pooled analysis.
Randomization	Randomization procedures are fully described in the previous primary publications which are correctly referenced in this pooled analysis. Randomization in both double-blind trials was accomplished through an interactive web-based response system.
Blinding	As noted in the paper, EASE LID and EASE LID 3 were both double-blind clinical trials. All patients, study site personnel, raters, the sponsor, and contract research organization staff were blinded to group assignment. EASE LID 2 was open-label.

Reporting for specific materials, systems and methods

We require information from authors about some types of materials, experimental systems and methods used in many studies. Here, indicate whether each material, system or method listed is relevant to your study. If you are not sure if a list item applies to your research, read the appropriate section before selecting a response.

Materials & experimental systems

n/a	Involved in the study	
☒	☐	Antibodies
☒	☐	Eukaryotic cell lines
☒	☐	Palaeontology and archaeology
☒	☐	Animals and other organisms
☐	☒	Human research participants
☐	☒	Clinical data
☒	☐	Dual use research of concern

Methods

n/a	Involved in the study	
☒	☐	ChIP-seq
☒	☐	Flow cytometry
☒	☐	MRI-based neuroimaging

Human research participants

Policy information about studies involving human research participants

Population characteristics	Participant characteristics are summarized in Table 1.
Recruitment	Recruitment was at specialist sites and was primarily based on the presence of dyskinesia.
Ethics oversight	Full ethics approval information is given in the prior primary publications. All three trials were conducted in compliance with the Declaration of Helsinki and International Conference on Harmonization (ICH) Good Clinical Practice (GCP) guidelines; all participating sites received institutional review board approval and all participants provided written informed consent before any procedures were performed.

Note that full information on the approval of the study protocol must also be provided in the manuscript.

Clinical data

Policy information about clinical studies

All manuscripts should comply with the ICMJE guidelines for publication of clinical research and a completed CONSORT checklist must be included with all submissions.

Clinical trial registration	NCT02136914, NCT02274766, NCT02202551
Study protocol	Protocols for the previously published trials are available at clinicaltrials.gov and on request

Data collection	EASE LID: May 7, 2014, and July 22, 2015 at 44 North American sites; EASE LID 3: 39 sites in the United States and Western Europe; EASE LID: 2 July 28, 2014, and March 10, 2016 at 67 sites in the United States and Western Europe
Outcomes	This was a pooled analysis of OFF time in the double-blind trials, which was prospectively collected in home diaries as a key secondary outcome measure. The MDS-UPDRS and CGI-C were also secondary outcomes. The MDS-UPDRS was the only PD rating instrument in the open-label trial, with MDS-UPDRS Part IV (items 4.1- 4.6) being the principal assessment of motor complications.
